# Supplementary material for: Mutations in HEADING DATE 1 affect transcription and cell wall composition in rice
Source: Plant Physiol. 2025 Mar 27;197(4):kiaf120. doi: 10.1093/plphys/kiaf120 (PMC12022608; doi:10.1093/plphys/kiaf120)
Supplement: kiaf120_Supplementary_Data [file kiaf120_supplementary_data.zip › SUPP FIGS Fabio Fornara.pdf]

Supplementary Figure S1

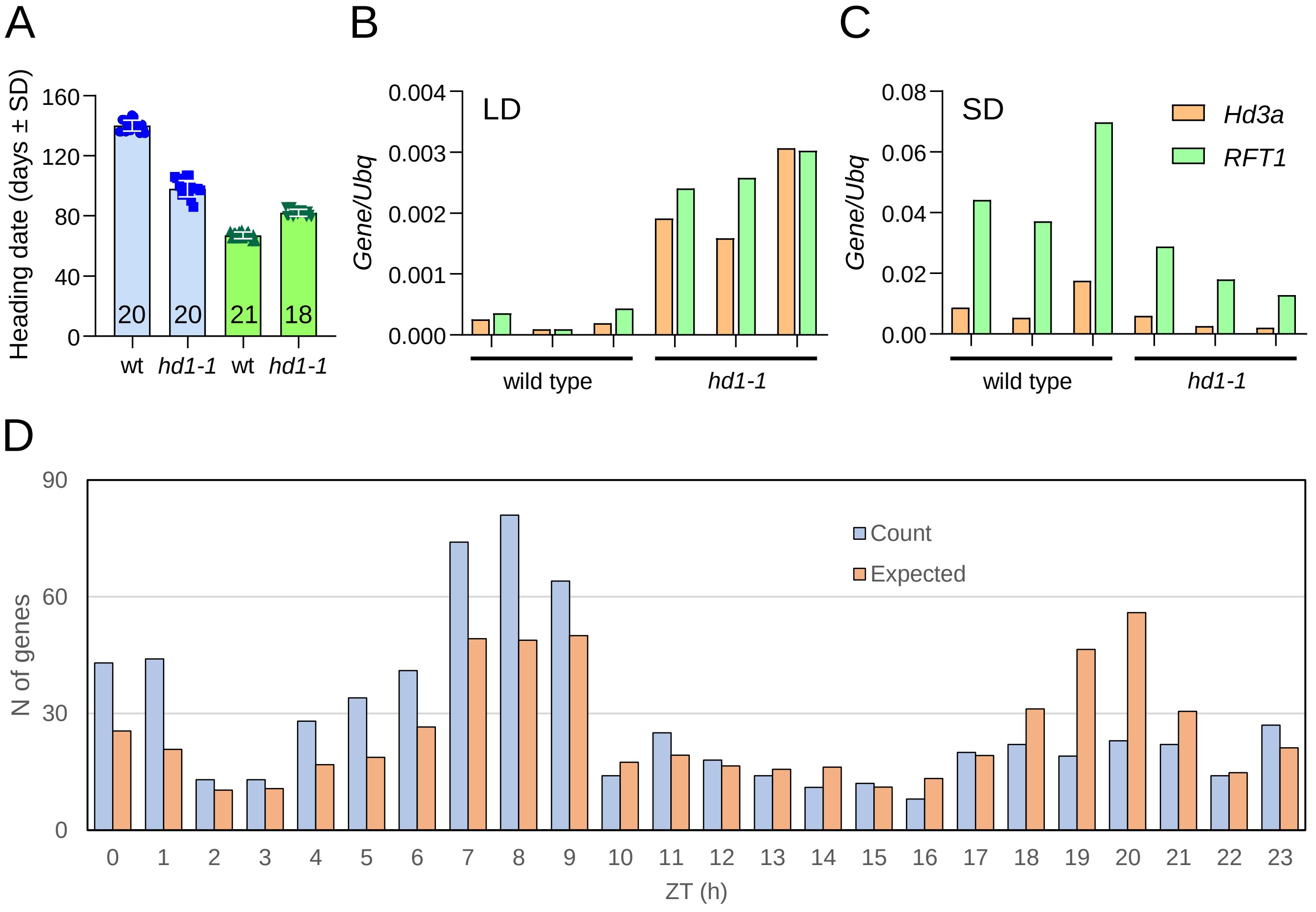

Supplementary Figure S1. RNA sequencing controls and phase enrichment of DE genes. **A**, flowering time of Nipponbare wild type and *hd1-1* mutants under LD (blue) and SD (green), expressed in days ± standard deviation. Each symbol corresponds to one plant. The number of plants scored is indicated in the histograms. **B-C**, expression of *Hd3a* and *RFT1* under LD (**B**) and SD (**C**) in the samples used for RNA sequencing. The three groups correspond to biological replicates. **D**, results of phase enrichment analysis obtained using Phaser (http://phaser.mocklerlab.org/) with default parameters. The number of genes is plotted against the phase of expression. ZT, *Zeitgeber*.

Supplementary Figure S2

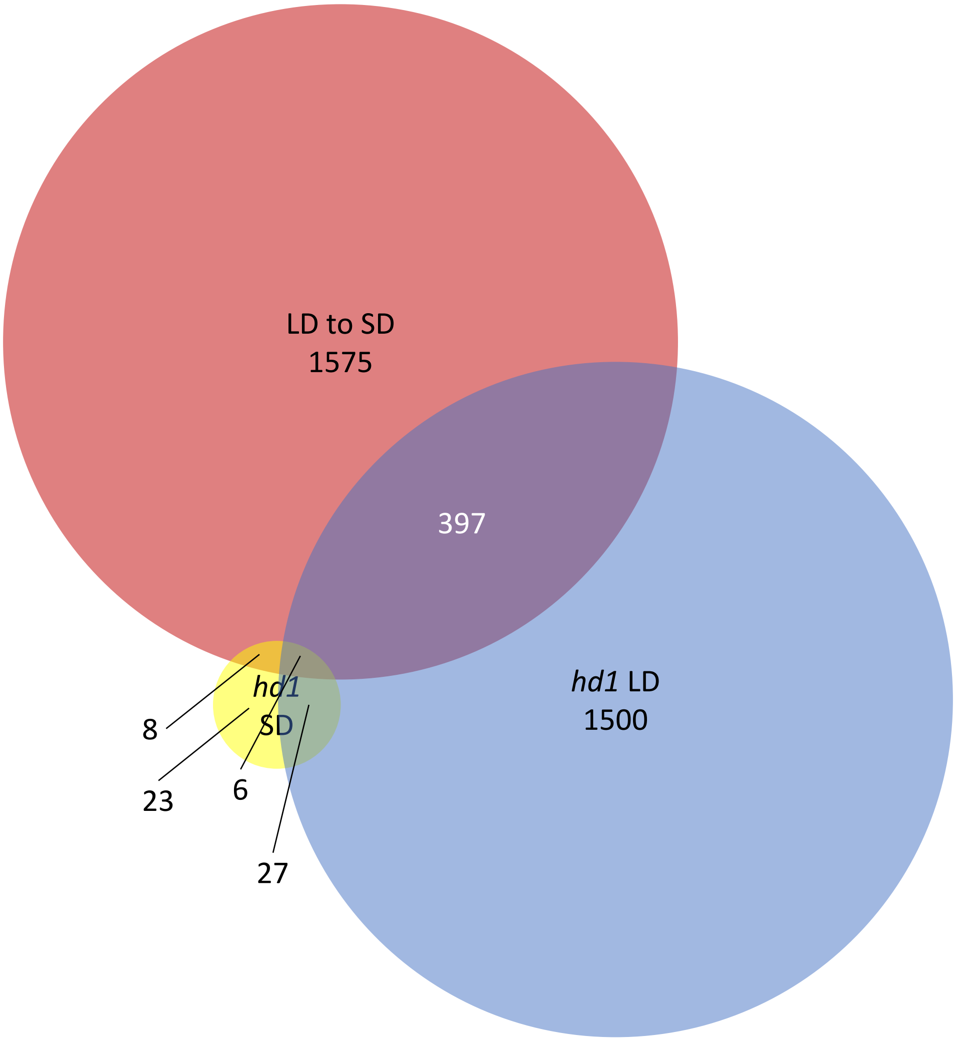

Supplementary Figure S2. Overlap between genes controlled by *Hd1* and the photoperiod. The Venn diagram shows the intersection between genes regulated by *Hd1* under LD (*hd1* LD), SD (*hd1* SD) and the shift from LD to SD (LD to SD). The size of the circles is proportional to the number of differentially expressed genes. The LD to SD dataset is from Galbiati *et al*., 2016. Genes were filtered for FDR≤0.01 and logFC≥|1.5|.

Supplementary Figure S3

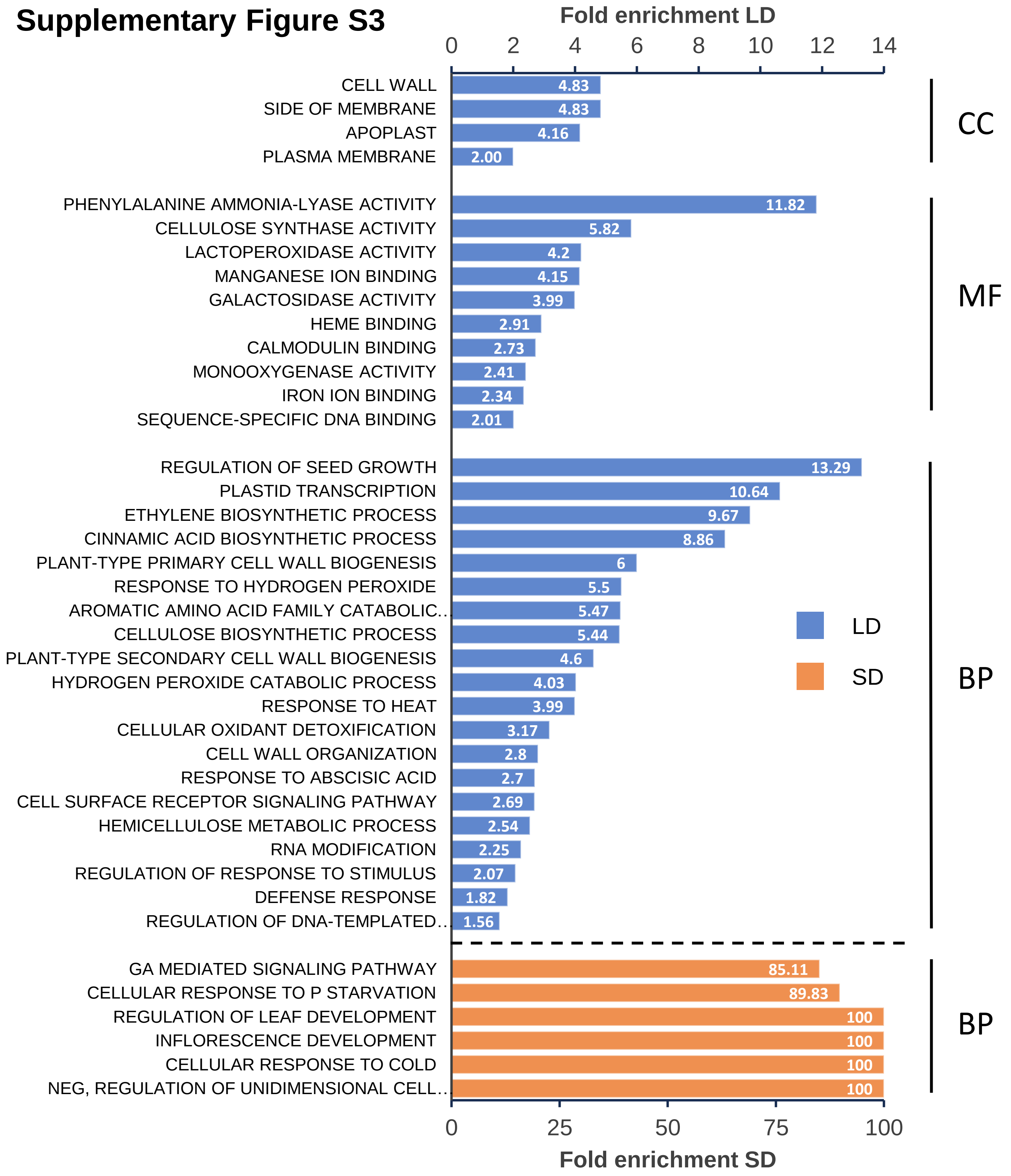

Supplementary Figure S3. Gene ontology categories enriched in the *hd1* transcriptomes. The graph shows categories significantly enriched more than 1.5 folds under LD and SD. Enrichments were performed using release 2024-01-17 of the Gene Ontology Resource database, with default settings. Input gene lists were filtered for FDR≤0.05 and $\log_2$FC≥|1.5|. Note that due to the small size of the SD dataset, few genes can cause a very high overrepresentation of some terms. Except for inflorescence development and GA mediated signaling pathway, categories are represented by *SPX1* and *SPX2* only, both of which are downregulated and control several developmental and physiological processes (Wang et al., 2014). CC, cellular compartment; MF, molecular function; BP, biological process.

Supplementary Figure S4

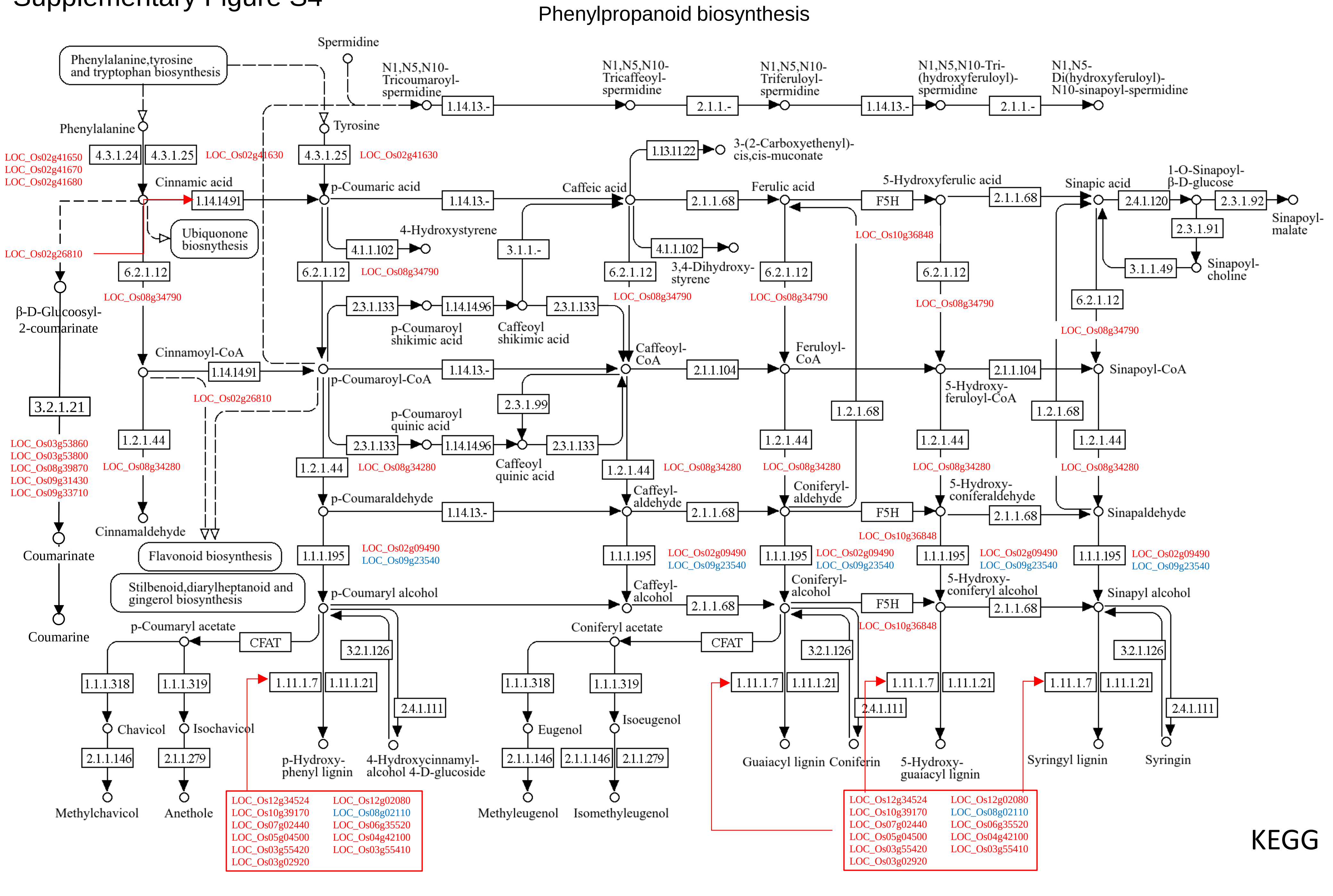

Supplementary Figure S4. Genes regulated by *Hd1* in the phenylpropanoid biosynthetic pathway. The image shows a scheme of the phenylpropanoid pathway as retrieved from the Kyoto Encyclopedia of Genes and Genomes (KEGG) database. The coumarine branch of the pathway was added manually. Gene identifiers are either red or blue when upregulated or downregulated under LD, respectively. Note that enzymes are often encoded by multiple genes.

Supplementary Figure S5

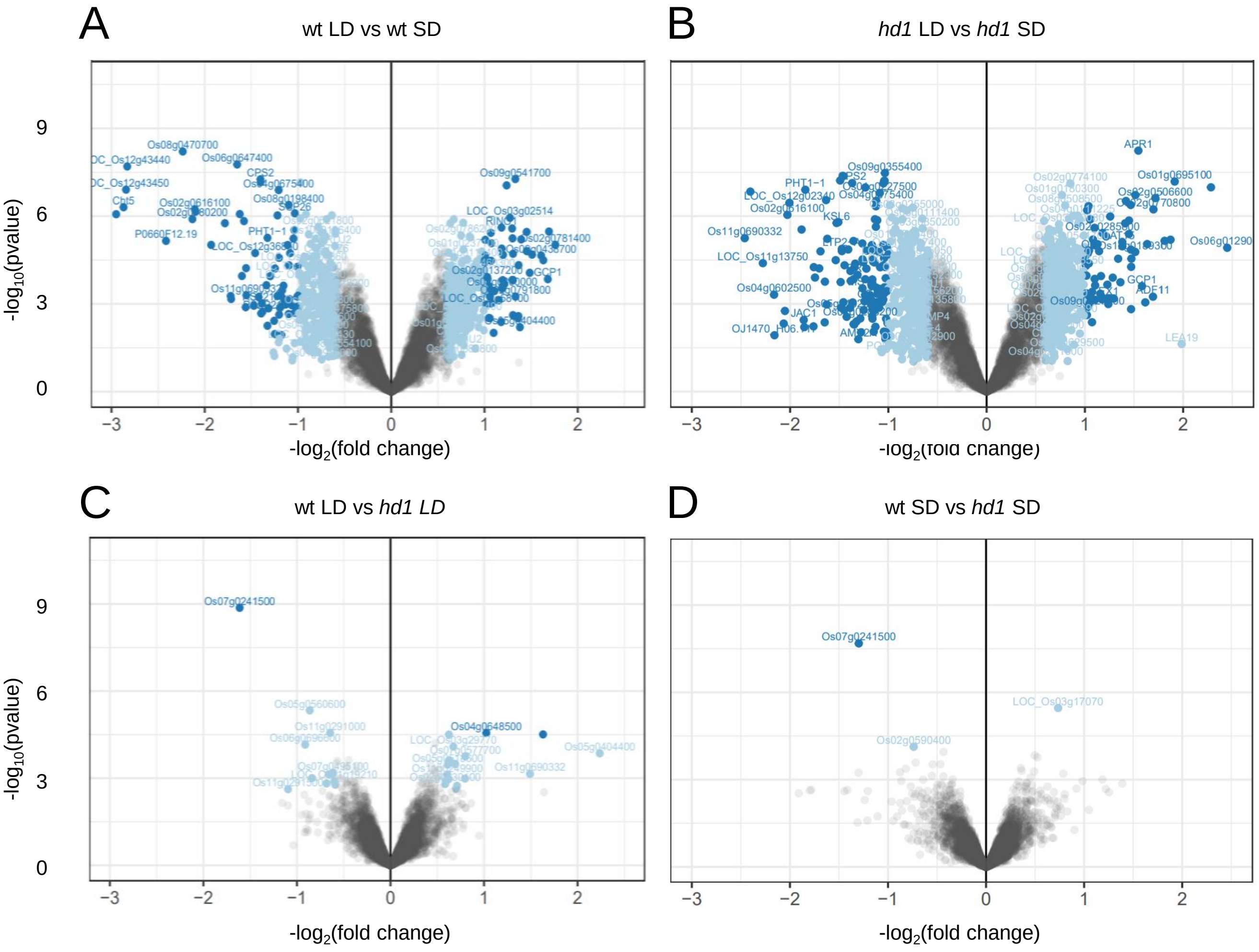

Supplementary Figure S5. The *hd1* leaf proteome under LD and SD. **A-D**, volcano plots showing differentially abundant proteins in the indicated comparisons. Expression coordinates are determined by -log$_2$FC and -log$_{10}$(p-value). Blue indicates protein hits (fdr<0.05 and absolute fold-change>2); light blue indicates protein candidates (fdr<0.2 and absolute fold-change>1.5); grey indicates proteins not significantly different between genotypes or treatments. Note that differences are mostly detected between photoperiods.

Supplementary Figure S6

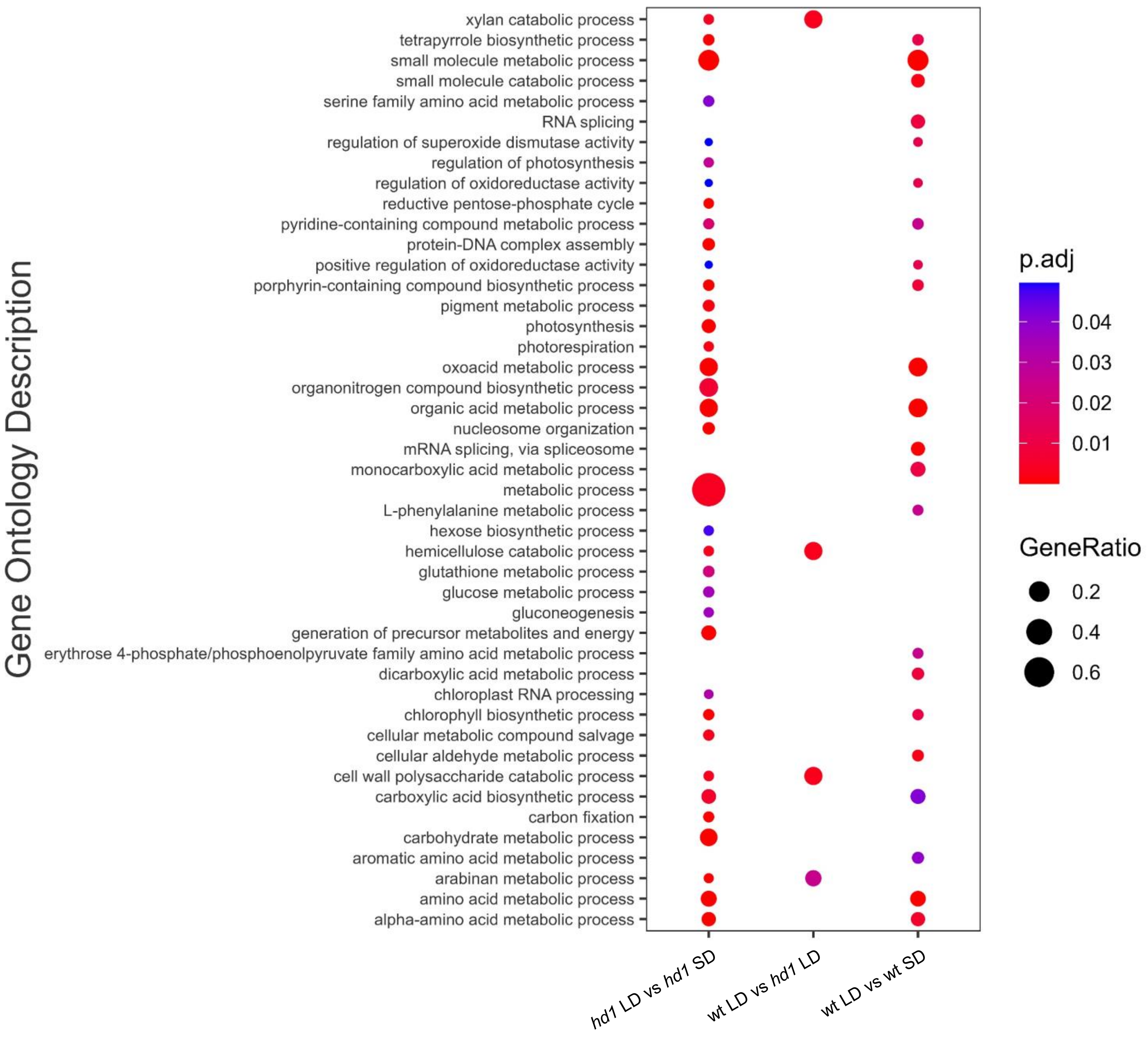

Supplementary Figure S6. Ontological categories of differentially expressed proteins. The graph shows gene ontology categories enriched in the lists of differentially abundant proteins, in the indicated comparisons. The size of the circles indicates the extent of enrichment; the color indicates the adjusted p-value.
