# Supplementary material for: Mutations in HEADING DATE 1 affect transcription and cell wall composition in rice
Source: Plant Physiol. 2025 Mar 27;197(4):kiaf120. doi: 10.1093/plphys/kiaf120 (PMC12022608; doi:10.1093/plphys/kiaf120)
Supplement: kiaf120_Supplementary_Data [file kiaf120_supplementary_data.zip › Supplementary Table 4 Fabio Fornara.docx]

**Supplementary table 4.** List of antibodies used in cell wall analysis.

| **Code** | **Epitope** | **Isotype** | **References** |
| --- | --- | --- | --- |
| JIM5 | Paritally methyl esterified homogalacturonan | IgG | (Knox et al., 1990; Clausen et al., 2003) |
| JIM7 | Methyl esterified homogalacturonan | IgA | (Knox et al., 1990; Clausen et al., 2003) |
| LM18 | Partially methyl esterified homogalacturonan | IgG | (Verhertbruggen et al., 2009) |
| LM19 | Unesterified homogalacturonan | IgM | (Verhertbruggen et al., 2009; Marcus et al., 2010; Yang et al., 2023) |
| LM20 | Methyl esterified homogalacturonan | IgM | (Verhertbruggen et al., 2009; Yang et al., 2023) |
| LM7 | Partially methyl esterified homogalacturonan | IgM | (Willats et al., 2001; Clausen et al., 2003) |
| RU2 | backbone of RG-I | IgM | (Ralet et al., 2010) |
| RU1 | backbone of RG-I | IgG | (Ralet et al., 2010) |
| LM5 | beta-1,4-galactan | IgG | (Jones et al., 1997; Andersen et al., 2016) |
| LM6 | alpha-1,5-arabinan | IgG | (Willats et al., 2001; Verhertbruggen et al., 2009) |
| LM16 | galactosylated RG-I | IgM | (Verhertbruggen et al., 2009) |
| LM21 | beta-1,4-mannan | IgM | (Marcus et al., 2010) |
| LM15 | Xylosyl residues of xyloglucan | IgG2c | (Marcus et al., 2008) |
| LM24 | Galactosyl residues of xyloglucan | IgG2a | (Pedersen et al., 2012) |
| LM25 | Xylosyl/galactosyl residues | IgM | (Pedersen et al., 2012) |
| LM11 | Beta-1,4-xylan | IgM | (McCartney et al., 2005) |
| LM23 | Xylosyl residues | IgM | (Manabe et al., 2011; Pedersen et al., 2012) |
| JIM8 | Arabinogalactan-protein (AGP) | IgG2c | (Knox et al., 1991; Moller et al., 2008; Wilkinson et al., 2017) |
| JIM13 | Arabinogalactan-protein (AGP) | IgM | (Knox et al., 1991; Moller et al., 2008; Wilkinson et al., 2017) |
| JIM15 | Arabinogalactan-protein (AGP) | IgM | (Knox et al., 1991; Moller et al., 2008; Wilkinson et al., 2017) |
| JIM16 | Galactosyl residue of AGP | IgM | (Knox et al., 1991; Moller et al., 2008; Wilkinson et al., 2017) |
| LM2 | Glucuronosyl residue of AGP | IgM | (Smallwood et al., 1996) |
| BS400-2 | (1-3)-beta-D-glucan | IgG | (Meikle et al., 1991) |
| BS400-3 | (1-3; 1-4)-beta-glucans | IgG | (Meikle et al., 1994) |
| CBM3a | cellulose (crystalline) | IgG | (Tormo et al., 1996) |

**References**

**Andersen MCF, Boos I, Marcus SE, Kračun SK, Rydahl MG, Willats WGT, Knox JP, Clausen MH** (2016) Characterization of the LM5 pectic galactan epitope with synthetic analogues of β-1,4-D-galactotetraose. Carbohydr Res. doi: 10.1016/j.carres.2016.10.012

**Clausen MH, Willats WGT, Knox JP** (2003) Synthetic methyl hexagalacturonate hapten inhibitors of anti-homogalacturonan monoclonal antibodies LM7, JIM5 and JIM7. Carbohydr Res. doi: 10.1016/S0008-6215(03)00272-6

**Jones L, Seymour GB, Knox JP** (1997) Localization of pectic galactan in tomato cell walls using a monoclonal antibody specific to (1→4)-β-D-galactan. Plant Physiol. doi: 10.1104/pp.113.4.1405

**Knox JP, Linstead PJ, Cooper JPC, Roberts K** (1991) Developmentally regulated epitopes of cell surface arabinogalactan proteins and their relation to root tissue pattern formation. The Plant Journal. doi: 10.1046/j.1365-313x.1991.t01-9-00999.x

**Knox JP, Linstead PJ, King J, Cooper C, Roberts K** (1990) Pectin esterification is spatially regulated both within cell walls and between developing tissues of root apices. Planta. doi: 10.1007/BF00193004

**Manabe Y, Nafisi M, Verhertbruggen Y, Orfila C, Gille S, Rautengarten C, Cherk C, Marcus SE, Somerville S, Pauly M, et al** (2011) Loss-of-function mutation of REDUCED WALL ACETYLATION2 in Arabidopsis leads to reduced cell wall acetylation and increased resistance to Botrytis cinerea. Plant Physiol. doi: 10.1104/pp.110.168989

**Marcus SE, Blake AW, Benians TAS, Lee KJD, Poyser C, Donaldson L, Leroux O, Rogowski A, Petersen HL, Boraston A, et al** (2010) Restricted access of proteins to mannan polysaccharides in intact plant cell walls. Plant Journal. doi: 10.1111/j.1365-313X.2010.04319.x

**Marcus SE, Verhertbruggen Y, Hervé C, Ordaz-Ortiz JJ, Farkas V, Pedersen HL, Willats WG, Knox JP** (2008) Pectic homogalacturonan masks abundant sets of xyloglucan epitopes in plant cell walls. BMC Plant Biol. doi: 10.1186/1471-2229-8-60

**McCartney L, Marcus SE, Knox JP** (2005) Monoclonal antibodies to plant cell wall xylans and arabinoxylans. Journal of Histochemistry and Cytochemistry. doi: 10.1369/jhc.4B6578.2005

**Meikle PJ, Bonig I, Hoogenraad NJ, Clarke AE, Stone BA** (1991) The location of (1→3)-β-glucans in the walls of pollen tubes of Nicotiana alata using a (1→3)-β-glucan-specific monoclonal antibody. Planta. doi: 10.1007/BF00194507

**Meikle PJ, Hoogenraad NJ, Bonig I, Clarke AE, Stone BA** (1994) A (1→3,1→4)‐β‐glucan‐specific monoclonal antibody and its use in the quantitation and immunocytochemical location of (1→3,1→4)‐β‐glucans. The Plant Journal. doi: 10.1046/j.1365-313x.1994.5010001.x

**Moller I, Marcus SE, Haeger A, Verhertbruggen Y, Verhoef R, Schols H, Ulvskov P, Mikkelsen JD, Knox JP, Willats W** (2008) High-throughput screening of monoclonal antibodies against plant cell wall glycans by hierarchical clustering of their carbohydrate microarray binding profiles. Glycoconj J. doi: 10.1007/s10719-007-9059-7

**Pedersen HL, Fangel JU, McCleary B, Ruzanski C, Rydahl MG, Ralet MC, Farkas V, Von Schantz L, Marcus SE, Andersen MCF, et al** (2012) Versatile high resolution oligosaccharide microarrays for plant glycobiology and cell wall research. Journal of Biological Chemistry. doi: 10.1074/jbc.M112.396598

**Ralet MC, Tranquet O, Poulain D, Moïse A, Guillon F** (2010) Monoclonal antibodies to rhamnogalacturonan I backbone. Planta. doi: 10.1007/s00425-010-1116-y

**Smallwood M, Yates EA, Willats WGT, Martin H, Knox JP** (1996) Immunochemical comparison of membrane-associated and secreted arabinogalactan-proteins in rice and carrot. Planta. doi: 10.1007/BF00620063

**Tormo J, Lamed R, Chirino AJ, Morag E, Bayer EA, Shoham Y, Steitz TA** (1996) Crystal structure of a bacterial family-III cellulose-binding domain: A general mechanism for attachment to cellulose. EMBO Journal. doi: 10.1002/j.1460-2075.1996.tb00960.x

**Verhertbruggen Y, Marcus SE, Haeger A, Verhoef R, Schols HA, McCleary B V., McKee L, Gilbert HJ, Knox JP** (2009) Developmental complexity of arabinan polysaccharides and their processing in plant cell walls. Plant Journal. doi: 10.1111/j.1365-313X.2009.03876.x

**Wilkinson MD, Tosi P, Lovegrove A, Corol DI, Ward JL, Palmer R, Powers S, Passmore D, Webster G, Marcus SE, et al** (2017) The Gsp-1 genes encode the wheat arabinogalactan peptide. J Cereal Sci. doi: 10.1016/j.jcs.2017.02.006

**Willats WGT, Orfila C, Limberg G, Buchholt HC, Van Alebeek GJWM, Voragen AGJ, Marcus SE, Christensen TMIE, Mikkelsen JD, Murray BS, et al** (2001) Modulation of the degree and pattern of methyl-esterification of pectic homogalacturonan in plant cell walls: Implications for pectin methyl esterase action, matrix properties, and cell adhesion. Journal of Biological Chemistry. doi: 10.1074/jbc.M011242200

**Yang X, Wilkinson LG, Aubert MK, Houston K, Shirley NJ, Tucker MR** (2023) Ovule cell wall composition is a maternal determinant of grain size in barley. New Phytologist. doi: 10.1111/nph.18714
