# Supplementary material for: Mutations in HEADING DATE 1 affect transcription and cell wall composition in rice
Source: Plant Physiol. 2025 Mar 27;197(4):kiaf120. doi: 10.1093/plphys/kiaf120 (PMC12022608; doi:10.1093/plphys/kiaf120)
Supplement: kiaf120_Supplementary_Data [file kiaf120_supplementary_data.zip › Supplementary Table 5 Fabio Fornara.docx]

**Supplementary Table 5**. Primers used in this study.

| **Target locus** | **Sequence 5’ – 3’** | **Primer name** |
| --- | --- | --- |
| *OsPAL4* (LOC_Os02g41680) | GATTAGCTGAATAACAGTGGAG | Os_1430 |
|  | GTTGCATGCTTGCCGCTATG | Os_1431 |
| *OsCAD8B* (LOC_ Os09g23540) | CCGCCTAGCTTGCTTGTTGT | Os_1432 |
|  | CTCCATTTCGTAGCTAGTTTGC | Os_1433 |
| *OsPAL1* (LOC_Os02g41630) | TAGCTGCTCTGCTCAGCTTG | Os_1824 |
|  | GATGCATTGAGCTGAAGACG | Os_1825 |
| *OsPAL2* (LOC_Os02g41650) | AAAAATGCAAAACAAGAAGACG | Os_1828 |
|  | TCATCACCATGTTCATCTAGACA | Os_1829 |
| *Hd1* coding sequence (LOC_Os06g16370) | GATGTCCCTTCACTTCAGCTC | Os_0554 |
|  | GGCCTTCTTCTTCTCCCTGT | Os_0023 |
| *Hd1* 3’ UTR | GATGTCCCTTCACTTCAGCTC | Os_0554 |
|  | CGATGTTATTGCTACTCAGTAAG | Os_0568 |
| *UBIQUITIN* (LOC_Os06g46770) | GACAACGTGAAGGCGAAGA | Os_1293 |
|  | CACCAGGTGGAGTGTGGAC | Os_1294 |
| *Hd3a* (LOC_Os06g06320) | CGATCTGCTGCATGCTCAC | Os_1287 |
|  | CCTTAGCCTTGCTCAGCTATTT | Os_1288 |
| *RFT1* (LOC_Os06g06300) | TGGGTTAGCTGACCTAGATTCAA | Os_1285 |
|  | GCCGGCCATGTCAAATTA | Os_1286 |
| *OsPAL4* promoter I | GGAACATCCCTAAATGGAACG | Os_1495 |
|  | CGGTCTTCTCGTGGTTTCTC | Os_1496 |
| *OsPAL4* promoter II | CTCTCGGTCAAGTCCATGCT | Os_1493 |
|  | TGGCAACTGCTCTTCAGATG | Os_1494 |
| *OsPAL4* promoter III | ATGTCGCCGAACAAGTCTCT | Os_1491 |
|  | CAGGTCTCGCTGATATGTTCC | Os_1492 |
| *OsPAL4* promoter IV | CAACCACCACACAGCCACAG | Os_1489 |
|  | CGTCGCGGAGTAGGAGAGAA | Os_1490 |
| *Hd3a* promoter | CCACACGTACAGGAAGACGATG | Os_614 |
|  | GAGAGTGAGATGGCCGCTTT | Os_615 |
